# Oral Granular Cell Tumor: A Case Report with Emphasis on Pseudoepitheliomatous Hyperplasia in Oral Lesions

**DOI:** 10.30476/dentjods.2023.98784.2108

**Published:** 2024-03-01

**Authors:** Saede Atarbashi-Moghadam, Ali Lotfi, Parsa Eftekhari-Moghadam

**Affiliations:** 1 Dept. of Oral and Maxillofacial Pathology, School of Dentistry, Shahid Beheshti University of Medical Sciences, Tehran, Iran; 2 Research Center, Dental School Shahid Beheshti University of Medical Sciences, Tehran, Iran

**Keywords:** Granular cell tumor, Oral cavity, Tongue, S100

## Abstract

A granular cell tumor (GCT) is an unusual benign mesenchymal neoplasm with Schwann cells origin. The most common site is the dorsum of the tongue. It has a striking tendency to occur in females and is more frequent in adult patients. GCT typically shows an asymptomatic, slow-growing, single nodule. Histopathologically, it reveals a proliferation of polygonal cells with granular cytoplasm penetrating the adjacent muscles. In some cases, the overlying epithelium demonstrates pseudoepitheliomatous hyperplasia (PEH), which can complicate its precise diagnosis and may mimic squamous cell carcinoma (SCC). This paper presents a 58-year-old woman with a chief complaint of painless mass on the dorsal of the tongue for two years. The lesion was pink and circumscribed with firm consistency measuring 1×1cm. The surface of the lesion was intact. Microscopic examination demonstrated unencapsulated sheets of large, polygonal cells with abundant eosinophilic, granular cytoplasm, and vesicular nuclei. The overlying epithelium showed florid PEH and keratin pearl formation. S100 protein was positive diffusely. The diagnosis of oral GCT was made. Though GCT is a non-aggressive lesion, it may be confused with SCC due to florid PEH and keratin pearl formation. Although PEH is a neglected topic among oral pathologists, it is of great importance in the field of research. Diagnosis can sometimes be problematic because they mimic other lesions. The pathogenesis of PEH is still uncertain. Therefore, familiarity with these characteristics and determining the cause of the PEH leads to correct treatment. This article intends to raise the insight of oral pathologists about PEH in oral lesions.

## Introduction

Granular cell tumor (GCT) is an uncommon benign connective tissue neoplasm with Schwann cells origin and is S100 positive [ 1 ]. GCT, together with schwannoma, neurofibroma, and traumatic neuroma, is included in the group of "peripheral nerve tumors" [ 2 ]. The difference between these neoplasms is in the proliferation and organization of cells. GCT tends to occur in adult women. This may be related to hormonal factors, preferring the differentiation of stem cells into Schwann cells in females. This theory has also been suggested in other nervous system neoplasms [ 1 ]. It characteristically shows an asymptomatic, slow-growing, single nodule located mainly in the tongue, followed by lips [ 1 ]. Multiple GCT, including oral and extraoral sites and variants associated with syndromes such as Noonan syndrome, has also been reported [ 1 , 3 ]. It may occur in the skin, soft tissues, breast, and lungs, but the head and neck region constitutes over 50% of cases, with the tongue being the most common location [ 4 ]. The treatment of choice is surgical excision, with a low recurrence rate of about 2.4%, associated with poor deep-margin excision [ 1 , 3 - 4 ]. Pseudoepitheliomatous hyperplasia (PEH) is seen in about one-third of cases of GCT, which can therefore be a distinctive microscopic characteristic feature of this tumor compared to other oral lesions [ 1 , 5 ]. PEH shows a tongue-like epithelial extension of the epithelium in underlying connective tissue and is a histopathologic term rather than a specific disease entity [ 6 ]. Its other names include pseudo carcinomatous hyperplasia, invasive acanthosis, verrucoid epidermal hyperplasia, and carcinomatoid hyperplasia [ 7 ]. Diagnosis can sometimes be problematic because they mimic other lesions. It may be challenging to differentiate lesions exhibiting PEH from invasive squamous cell carcinoma (SCC), particularly in small biopsies, inadequate excision, inappropriate orientation, and dense inflammatory infiltration [ 6 ]. 

This paper presents a 58-year-old female patient with tongue GCT with florid PEH and emphasizes the etiologic factors of PEH in oral lesions.

## Case Presentation

**Figure 1:**
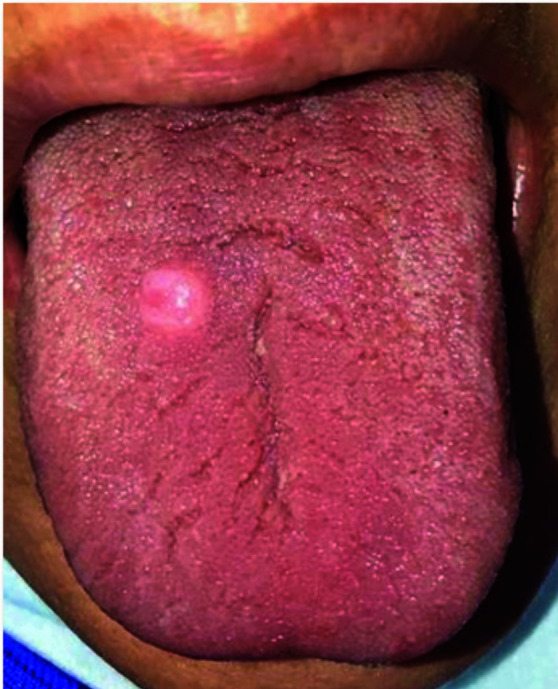
A pink and circumscribed firm mass on the dorsum of the tongue

**Figure 2:**
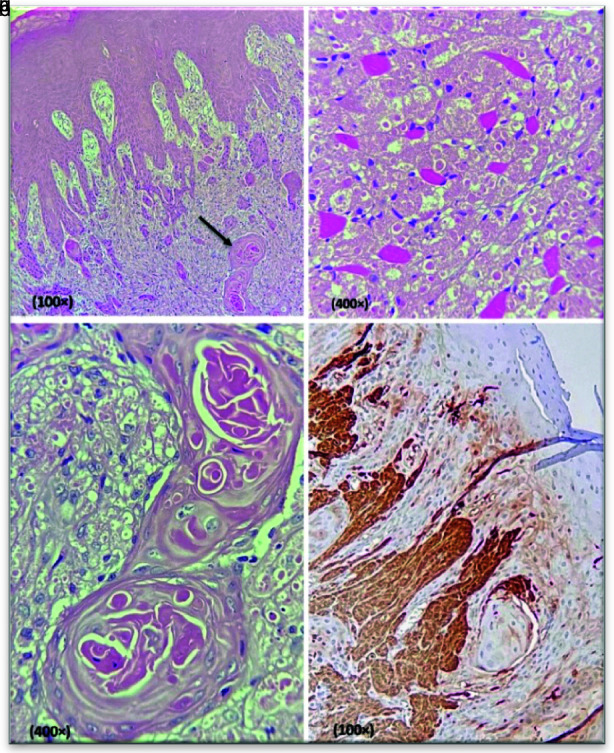
**a:** Microscopic sections show a proliferation of polygonal cells with granular cytoplasm penetrating the adjacent muscles. The overlying epithelium demonstrates florid PEH and keratin pearl formation (black arrow) (H&E); **b:** Granular cells (H&E); **c:** Keratin pearls (H&E); **d:** The granular cells are positive diffusely for S-100 protein (IHC)

A 58-year-old woman with a chief complaint of painless mass on the dorsal of the tongue with two years duration was referred to a private dental clinic (Tehran, Iran) in January 2023. The lesion was pink and circumscribed with firm consistency measuring 1×1cm. The surface of the lesion was intact (Figure 1). She mentioned that the surface of the lesion was traumatized a few months ago, but it was healed. A provisional diagnosis of a benign mesenchymal tumor or reactive lesion such as focal fibrous hyperplasia was made, and an excisional (punch) biopsy was done under local anesthesia. Histopathologic examination showed a benign mesenchymal neoplasm composed of unencapsulated sheets of large, polygonal cells with abundant eosinophilic, granular cytoplasm, and vesicular nuclei. Transitioning from normal adjacent skeletal muscle fibers to granular cells was also evident. The lesion was covered with para to orthokeratinized stratified squamous epithelium with florid PEH and keratin pearl formation. Immunohistochemical staining for S-100 protein was diffusely positive (Figure 2). According to the information above, the diagnosis of GCT was performed. The patient is under follow-up.

## Discussion

Some clinicopathological feature recommends a possible reactive origin in GCT. This theory would describe the absence of any report of malignant intraoral GCT and a low recurrence rate even with insufficient surgery [ 1 , 4 ]. On the other hand, due to a direct association between evolution time and lesion size, the neoplastic origin can be considered [ 1 ]. Histopathologically, GCT shows an unencapsulated proliferation of polygonal cells with granular cytoplasm that penetrate the adjacent muscles. The cell borders are frequently indistinct, which results in a syncytial appearance [ 4 - 5 ]. Groups of granular cells may envelop small nerve bundles [ 1 , 4 - 5 ]. GCT cells reveal a very low Ki-67 proliferation rate, indicating limited growth [ 5 ]. Though GCT shows positivity for CD57, CD56, PGP9.5, P75, and neuron-specific enolase (NSE), S100 protein remains a diagnostic marker [ 1 , 4 ]. In the oral cavity, the histopathologic differential diagnosis includes a granular cell odontogenic tumor, a granular cell variant of ameloblastoma, and congenital epulis, all of which are negative for S100 [ 8 ]. The GCT is best treated by conservative local excision. The recurrence rate is low even in cases where the tumor is not completely removed [ 3 ]. 

PEH is a microscopic reaction pattern to different stimuli, which comprises trauma, infection, inflammation, and neoplasia. PEH proliferates from the overlying epithelium and other skin adnexa [ 7 , 9 ]. It has been stated that PEH is detected frequently in mucosal surfaces rich in salivary glands, and they proposed a glandular origin for PEH [ 7 ]. PEH indicates some degree of epithelial thickening. The interface between epithelial and connective tissue is less clear, and the proliferating epithelial projections tend to anastomose, entrapping the stromal part [ 7 ]. Hypergranulosis and ortho- or parakeratosis are frequently seen. Keratin pearl formation may be seen. Mitosis may be present but not abundant or atypical [ 10 ]. Oral conditions associated with PEH include blastomycosis, Wegener's granulomatosis, GCT, necrotizing sialometaplasia, pemphigus vegetans, median rhomboid glossitis, epulis fissuratum, chronic hyperplastic candidiasis, and lymphoproliferative disorders [ 7 , 10 ]. In addition, lesions that may reveal PEH include oral melanoma, intramucosal and Spitz nevus, oral submucous fibrosis, and actinomycosis [ 7 ]. 

Typically, the result of any physical or chemical injury is inflammation followed by the removal of devitalized tissue. Later, connective tissue proliferation and vascular tissue growth are seen. In the end, re-epithelization followed by fibrous maturation occurs. Any disturbance in this process shows a disorganized arrangement of normal epithelial structure exhibiting PEH [ 6 ]. The function of PEH is not clear. It is assumed that it occurs as a physiological response to skin injuries.

Moreover, it is supposed to act as a defensive reaction for transepithelial removal of foreign body material [ 1 , 9 ]. Neoplastic cells or inflammation release varying cytokines that cause the proliferation of overlying epithelium and PEH [ 6 ]. Chrysomali *et al*. [ 5 ] found that GCTs with PEH displayed a notable Ki-67 proliferation rate of the surface epithelium basal cells. They suggested that the proximity of the epithelium to the granular cells might have led to the up-regulation of basal cell proliferation. It may provide a reasonable description of the association of PEH with GCT [ 5 ]. Lafuente Ibáñez de Mendoza *et al*. [ 1 ] mentioned that GCT with PEH showed more than one year of evolution compared to non-PEH GCTs.

It may be challenging to differentiate lesions exhibiting PEH from invasive SCC [ 6 ]. Microscopic characteristics that favor SCC comprise nuclear atypia, -increased mitosis, individual cell keratinization, and deep invasion of epithelial nests into the stroma. In the present case report, the PEH was florid but superficial. Although keratin pearls were formed, no cell atypia was seen. Recognizing the underlying disease process, careful clinical examination, serial sections of the specimen or sometimes, additional biopsy may be mandatory to get a correct diagnosis [ 10 ]. In the present case of GCT, the small size of the lesion, the long duration, and the involvement of the tongue's dorsal surface all help differentiate this lesion from invasive SCC. Immunohistochemical staining may be helpful. SCC reveals increased expression of p53 and MMP-1, while E-cadherin displays less intense staining [ 10 ]. 

Intraosseous PEH related to chronic osteomyelitis and infected osteoradionecrosis have been reported. 

In addition, PEH associated with medication-related -osteonecrosis of the jaw (MRONJ), previously identified as bisphosphonate-related osteonecrosis of the jaw (BRONJ), has been described [ 11 - 12 ]. Bouquot *et al*. [ 12 ] mentioned that MRONJ-associated PEH must be ruled out before diagnosing carcinoma for these lesions. However, any squamous epithelium in the bone at the preceding oral SCC location would highly indicate residual or recurrent carcinoma [ 11 ]. Ide *et al*. [ 11 ] stated that although the degree of proliferation is variable, PEH is relatively common in bone. 

Informed consent was obtained from the patient for publishing her clinical photograph of the tongue.

## Conclusion

Although GCT is a non-aggressive lesion, it may be confused with SCC due to florid PEH and keratin pearl formation. Trauma, infection, inflammation, and neoplasia have been suggested in developing PEH. Accurate clinical examination, serial sections of the specimen, or sometimes another biopsy may be required to get a precise diagnosis. The absence of nuclear atypia and mitotic figures and the recognition of the underlying factors will help differentiate PEH from SCC. Familiarity with this phenomenon is essential for pathologists and prevents potential inappropriate treatment.
